# Antifungal Susceptibility Does Not Correlate With Fungal Clearance or Survival in AIDS-Associated Cryptococcal Meningitis

**DOI:** 10.1093/cid/ciaa1544

**Published:** 2020-10-14

**Authors:** Lucy O’Connor, Duong Van Anh, Tran Thi Hong Chau, Nguyen Van Vinh Chau, Lan Nguyen Phu Huong, Marcel Wolbers, Jeremy N Day

**Affiliations:** 1 Guy’s and St Thomas’ NHS Trust, United Kingdom; 2 Oxford University Clinical Research Unit, Ho Chi Minh City, Viet Nam; 3 Hospital for Tropical Diseases, Ho Chi Minh City, Viet Nam; 4 Centre for Tropical Medicine and Global Health, Nuffield Department of Medicine, University of Oxford, Oxford, United Kingdom

**Keywords:** cryptococcal meningitis, susceptibility testing, HIV, outcome, mortality

## Abstract

We investigated the value of susceptibility testing in predicting response in AIDS-associated cryptococcal meningitis using clinical isolates from a randomized controlled trial of antifungal treatment (amphotericin monotherapy, amphotericin with flucytosine, or amphotericin with fluconazole). We found no correlation between antifungal susceptibility and either early or late survival, or fungal clearance.

*Cryptococcus neoformans* causes devastating meningitis and 15% of AIDS-related deaths globally. It is an environmental saprophyte acquired through inhalation; azole-resistance may reflect exposure to agricultural pesticides. WHO guidelines, available at https://www.who.int/hiv/pub/guidelines/cryptococcal-disease/en/, recommend induction therapy with amphotericin and flucytosine, which delivers improved cerebrospinal fluid (CSF) sterilization and survival [1, 2]. Flucytosine is unaffordable for countries with the greatest disease burden. Consequently, many patients receive inferior treatment with amphotericin, alone or with fluconazole [1].

Broth microdilution is the current standard method for antifungal susceptibility testing (AST) of yeasts, as outlined by the Clinical Laboratory Standards Institute (CLSI) and European Committee on Antimicrobial Susceptibility Testing (EUCAST) [3]. There is substantial deviation from standardized methods in published literature, with varying media, incubation times, and methods of end-point determination. The Sensititre YeastOne system (Thermo Fisher Scientific, UK) is a commercially available broth microdilution method for AST with good essential agreement with CLSI and EUCAST methods [3]. It has the advantages of ease of use and interpretation via a colorimetric binary output, and consistency through central manufacturing.

There is little consensus on the value of AST in cryptococcal meningitis; few studies demonstrate any association between susceptibility and outcome [4–6], although attempts using modified methods have been more successful [6]. The variety of methods, lack of susceptibility breakpoints for *C. neoformans*, heterogeneity of induction regimens, and inconsistent assessment of baseline disease severity make these studies difficult to compare.

Previously, we reported the results of a randomized controlled trial (RCT) of induction therapy for AIDS-associated cryptococcal meningitis [1]. Here, we use isolates obtained at diagnosis in this study to determine the ability of AST to predict therapeutic response.

## METHODS

### Patient Population

We enrolled 299 patients into an open-label RCT of antifungal therapy for AIDS-associated cryptococcal meningitis at a single center in Vietnam between 2005 and 2010. Detailed trial methodology has been described previously [1]. Patients received induction treatment with either amphotericin monotherapy (1 mg/kg/day for 4 weeks), amphotericin combined with flucytosine (100 mg/kg/day for 2 weeks), or amphotericin combined with fluconazole (400 mg twice daily for 2 weeks), followed by consolidation with fluconazole monotherapy (400 mg daily) until 10 weeks postrandomisation (see Supplementary Fig. 1).

### Fungal Isolates and Susceptibility Testing

CSF quantitative fungal counts were determined as previously described [1]. *Cryptococcus* isolates were cultured from CSF at randomization and archived via a full plate sweep with storage on beads (Pro-Lab Diagnostics, UK) at −80°C. For AST, isolates were revived on Sabouraud plates, a single colony selected and purified by culture, and the susceptibility of this single isolate to amphotericin, fluconazole, and flucytosine determined using Sensititre YeastOne as per the manufacturer’s instructions.

### Statistical Analysis

We analyzed the joint effect of the relevant drug Minimum Inhibitory Concentration (MIC) for patients on combination therapy and the effect of amphotericin MIC for patients on monotherapy. Primary outcome was survival until 70 days analyzed using Cox regression. Secondary outcomes were survival until 14 days and 6 months, and CSF fungal decline over the first 14 days (estimated from longitudinal measurements during that period and a linear mixed-effects model). The Cox model was also analyzed with adjustment for baseline fungal burden and Glasgow Coma Score (GCS), these factors being associated with worse outcome [1]. Data were included for all strains with a valid MIC result, and are presented for participants by trial arm in terms of the effect that a 2-fold increase in MIC has on the outcome measure of interest. We also defined isolates as fully sensitive or not, using breakpoints from published literature and as suggested in CLSI guidelines; MIC ≤0.512 µg/mL for amphotericin B, ≤4 µg/mL for flucytosine, ≤8 µg/mL for fluconazole at 72 hours [4]. All analyses were performed using R software version 2.13.1 (https://www.r-project.org/.).

## RESULTS

Of 299 study participants, 23 were excluded (no viable baseline isolate n = 9; inadequate growth by 72 hours n = 12; missing purity plate data, n = 2). Baseline characteristics of the primary analysis population are in Supplementary Table 1. Drug susceptibilities were similar between treatment arms (Supplementary Table 2); the range of susceptibilities is illustrated in Supplementary Figure 2.

### Primary Outcomes (Patient Survival)

Table 1 shows the estimated effect on survival of decreasing antifungal susceptibility by 70 days postrandomization, without adjustment for disease severity. There was no consistent trend in hazard ratios (HR) produced by the model. Due to the multiplicity of analyses, individual HR estimates and *P*-values should be interpreted with caution. The adjusted model produced similar results.

**Table 1.** The estimated effect, defined by hazard ratio (HR) and 95% confidence intervals, of a 2-fold increase in the MICs estimated at 72 hours for amphotericin, fluconazole, and flucytosine on survival outcomes at 14, 70, and 182 days postrandomisation for the primary analysis population and the effect on mycological outcome (the rate of decline of CSF fungal count, log10 Colony Forming Units/mL CSF/day)

	Group 1 Amphotericin (n = 92)		Group 2 Amphotericin and Flucytosine (n = 96)		Group 3 Amphotericin and Fluconazole (n = 88)	
	HR	*P* value	HR	*P* value	HR	*P* value
**Death by day 14**						
Effect on the rate of death per 2-fold increase in the MIC (95% CI) of:						
** Amphotericin**	0.64 (.28, 1.44)	.28	0.86 (.35, 2.13)	.75	0.69 (.27, 1.75)	.43
** Flucytosine**	-	-	0.70 (.33, 1.47)	.34	-	-
** Fluconazole**	-	-	-	-	1.23 (.63, 2.43)	.54
**Death by day 70**						
Effect on the rate of death per 2-fold increase in the MIC (95% CI) of:						
** Amphotericin**	0.94 (.65, 1.86)	.83	0.58 (.31, 1.09)	.09^a^	0.97 (.47, −2.01)	.94
** Flucytosine**	-	-	0.88 (.55, 1.39)	.58	-	-
** Fluconazole**	-	-	-	-	0.87 (.51, 1.49)	.61
**Death by 6 months (day 182)**						
Effect on the rate of death per2-fold increase in the MIC (95% CI) of:						
** Amphotericin**	1.10 (.65, 1.86)	.72	0.62 (.34, 1.11)	.11^b^	1.28 (.71, 2.30)	.42
** Flucytosine**	-	-	0.89 (.58, 1.39)	.62	-	-
** Fluconazole**	-	-	-	-	0.86 (.54, 1.36)	.51
**Change in CSF fungal decline in first 14 days (log10 CFU/mL of CSF per day) per 2-fold increase in MIC**						
Numbers in brackets are 95% Confidence Intervals						
	**Effect estimate**	***P* value**	**Effect estimate**	***P* value**	**Effect estimate**	***P* value**
** Amphotericin**	−0.01 (−.07, .04)	.59	0.02 (−.03, .07)	.40	0.00 (−.04, .04)	.95
** Flucytosine**	-	-	1.10 (.80, 1.49)	.63	-	-
** Fluconazole**	-	-	-	-	0.01 (−.02, .04)	.53

^a^When adjusted for baseline CSF log-quantitative fungal count, GCS below 15 and *Cryptococcus* genotype at recruitment the results for a 2-fold increase in 72 hour MIC for amphotericin in group 2 were 0.55 (95% CI, .30–1.01), *P* value .053.

^b^When adjusted as before, results for a 2-fold increase in 72 hour MIC for amphotericin in group 2 were 0.58 (95% CI, .33–1.03), *P* value .06.

The Kaplan-Meier curves in Supplementary Figure 3 illustrate the estimated effect of antifungal susceptibility on time to death up to 6 months when patients’ isolates are categorized as either “fully sensitive” or “nonsusceptible” (Supplementary Table 3). We found no evidence that this categorization affected risk of death, including in an exploratory analysis of patients with high fungal loads (defined as >6 × 10^6^ colony forming units/mL CSF).

### Secondary Outcomes

We found no evidence that antifungal susceptibility affected either the early (day 14) or late (6 month) hazard of death (Table 1). The adjusted model produced similar results for all outcome measures. We found no consistent effect of drug susceptibility on the rate of fungal clearance from CSF for any of the 3 drugs tested.

## Discussion

The most effective induction regimen for cryptococcal meningitis, amphotericin combined with flucytosine, results in mortality rates of 15% to 40% [1, 2]. Trial data suggest that amphotericin accelerates CSF fungal clearance, but amphotericin toxicity contributes to mortality when therapy continues for more than 1 week [2]. Given the high mortality rate, toxicity, and cost of combination induction therapy, the ability to predict a patient’s response to antifungal therapy at diagnosis would enable optimization of the limited therapeutic options available [6]. However, we found no evidence that AST, measured using Sensititre YeastOne, can help guide treatment choices in cryptococcal meningitis.

Some small studies report an association between *Cryptococcus* susceptibility and survival. Witt and colleagues [7] found that fluconazole susceptibility was an independent predictor of treatment outcome (survival at 10 weeks with sterile CSF) in HIV-associated disease; however, they failed to demonstrate this association using the CLSI macrotiter method, used an amphotericin-free treatment regimen, and did not adjust for baseline disease severity. A small, retrospective study by Lee (n = 46) found an association between fluconazole susceptibility and survival, but none for amphotericin B or flucytosine. This study included a heterogeneous patient population managed with multiple treatment regimens, which were not adjusted for in the statistical analysis, potentially confounding results [6].

In contrast, larger studies, including ours, with more robust sampling and less selection bias by analysis of subsets of RCTs, fail to show an association between susceptibility and survival [4, 5, 8–10]. The strengths of our study are its size, the randomized allocation of induction therapy (removing bias in treatment selection), standardization of drug formulations, care delivery within a single institution, and AST within a single laboratory. We found no evidence that AST of isolates at the point of diagnosis predicts mycological response or survival, even in patients with high fungal burdens. This was true for all 3 key antifungal drugs (amphotericin, flucytosine, and fluconazole), for survival at both early (14 day) and late (70, 182 day) time-points, and following adjustment for baseline factors associated with severity, including fungal burden. We must conclude that AST has no utility in optimizing therapy for patients with a first presentation of cryptococcal meningitis.

There are several possible explanations for the poor correlation between antifungal susceptibility of *C. neoformans* and therapeutic outcomes. These include significant differences between in vivo infection and in vitro AST environments. *Cryptococcus neoformans* variably expresses its phenotype in different models and culture systems, including virulence factors (melanization, polysaccharide capsule size, titan cell formation) which may influence susceptibility. Second, host–drug interactions may play a role in clinical response; amphotericin may have immunomodulatory effects promoting yeast clearance that cannot be reflected in vitro, and may be variably expressed in AIDS patients [6].

A potential weakness of our study is that we tested only single purified isolates from our patients. While the majority of immunosuppressed patients have infections from a single strain of *C. neoformans*, multiple strain infections may occur in up to 18%, challenging the concept of correlating outcome with the susceptibility of a single isolate [11]. A further potential weakness is that we tested only baseline isolates; *C. neoformans* displays the phenomenon of heteroresistance to azoles whereby a resistant subpopulation can emerge from within a predominantly susceptible single strain following azole exposure; this may contribute to disease relapse [12, 13].

In conclusion, we present robust data that AST of baseline isolates of *C. neoformans* in AIDS-associated cryptococcal meningitis does not correlate with survival or mycological clearance; it has no place in routine clinical use in first cases of cryptococcal meningitis. However, several aspects warrant further investigation. Our study enrolled only severely immunosuppressed patients, which may confound any effect of susceptibility on outcome; similar data should be generated for immunocompetent patients. Furthermore, because of the phenomenon of heteroresistance, AST may be more informative if measured following a few days of treatment [12].

## Supplementary Data

Supplementary materials are available at *Clinical Infectious Diseases* online. Consisting of data provided by the authors to benefit the reader, the posted materials are not copyedited and are the sole responsibility of the authors, so questions or comments should be addressed to the corresponding author.

ciaa1544_suppl_Supplementary_Appendix

## Notes

***Acknowledgments.*** The authors thank the patients and staff of the Hospital for Tropical Diseases.

***Financial support.*** Supported by the Wellcome Trust [077078/Z/05/A and 089276/Z/09/Z] and British Infection Society.

***Potential conflicts of interest.*** M. W. is currently an employee of Roche Pharma. The contributions to this manuscript are unrelated to this position and were conducted during earlier employment or in his personal capacity only. J. N. D. reports grants from Wellcome Trust and the British Infection Society during the conduct of the study. L. O. C., D. V. A., T. T. H. C., N. V. V. C. and L. N. P. H. report no potential conflicts of interest. All authors have submitted the ICMJE Form for Disclosure of Potential Conflicts of Interest. Conflicts that the editors consider relevant to the content of the manuscript have been disclosed.
