# Supplementary material for: Antifungal Susceptibility Does Not Correlate With Fungal Clearance or Survival in AIDS-Associated Cryptococcal Meningitis
Source: Clin Infect Dis. 2020 Oct 14;73(7):e2338–41. doi: 10.1093/cid/ciaa1544 (PMC8561241; doi:10.1093/cid/ciaa1544)
Supplement: ciaa1544_suppl_Supplementary_Appendix [file ciaa1544_suppl_Supplementary_Appendix.docx]

**Supplementary Appendix**

**Methods for Susceptibility Testing**

Minimum inhibitory concentrations (MICs) were estimated for all isolates after 72 hours incubation at 35°C. Isolates without growth at 35°C were incubated at 30°C, and MICs estimated as before. Candida krusei ATCC 6258 was used as a control strain.

**Statistical methods**

Quantitative fungal counts were log-transformed for all analyses. Prior to analysis, MICs below the limit of detection were replaced by (*min*/2), where *min* is the lowest concentration of antifungal agent in the Sensititre YeastOne system; MICs above the limit of detection were replaced by 2**max*, where *max* is the highest concentration of antifungal agent.

Analyses were adjusted for randomised treatment assignment and tested for interaction between MICs and trial treatment arms. Analyses comparing the survival effect of ‘susceptible’ and ‘non-susceptible’ isolates in a Cox regression model were performed both with and without adjustment for baseline CSF log-quantitative fungal count, Glasgow Coma Scale score (GCS) below 15 at recruitment (previously identified as independent predictors of outcome), and AFLP cluster (VN1gamma versus not VN1gamma) [1,2].

Time to fungal clearance was estimated with a cause-specific Cox regression model adjusted for baseline CSF log-quantitative fungal count.

**Results**

CSF log-quantitative fungal count and GCS below 15 at recruitment produced similar results.

**Supplementary Data**

**Supplementary Table 1.** Summary of baseline characteristics for the primary analysis population (n=276)

| **Characteristic** | **Group 1**  **Amphotericin**  **(n=92)** | **Group 2**  **Amphotericin and Flucytosine**  **(n=96)** | **Group 3**  **Amphotericin and Fluconazole**  **(n=88)** |
| --- | --- | --- | --- |
| Age – yr ^a^ |  |  |  |
| Median | 28 | 28 | 27 |
| Interquartile range | 25, 31 | 25, 33 | 24, 31 |
| Male sex – no. (%) | 76 (83) | 76 (79) | 75 (85) |
| Intravenous drug use – no./total no. (%) | 47/84 (56) | 45/90 (50) | 48/87 (55) |
| Glasgow Coma Scale score ^b^ |  |  |  |
| Median | 15 | 15 | 15 |
| Interquartile range | 13, 15 | 14, 15 | 15, 15 |
| CD4 count – cells/mm^3^ ^c^ |  |  |  |
| Median | 18 | 16 | 14 |
| Interquartile range | 8, 36 | 9, 27 | 8, 41 |
| CSF opening pressure - cmH_2_O ^d^ |  |  |  |
| Median | 27 | 32 | 24 |
| Interquartile range | 15, 40 | 19, 40 | 16, 40 |
| CSF white cell count – cells/mL ^e^ |  |  |  |
| Median | 34 | 26 | 25 |
| Interquartile range | 8, 86 | 7, 63 | 7, 84 |
| CSF yeast count – log_10_ CFU/mL ^f^ |  |  |  |
| Median | 5.95 | 5.82 | 5.74 |
| Interquartile range | 5.60, 6.49 | 4.70, 6.15 | 4.80, 6.23 |
| Weight – kg ^g^ |  |  |  |
| Median | 46 | 48 | 48 |
| Interquartile range | 42, 50 | 41, 50 | 45, 50 |

^a^Data were missing for 1 patient in group 3

^b^Data were missing for 2 patients in group 1, 1 in group 2 and 1 in group 3

^c^Data were missing for 23 patients in group 1, 24 in group 2, 23 in group 3

^d^Data were missing for 16 patients in group 1, 19 in group 2, 17 in group 3

^e^Data were missing for 9 patients in group 1, 10 in group 2, 10 in group 3

^f^Data were missing for 19 patients in group 1, 19 in group 2, 19 in group 3

^g^Data were missing for 2 patients in group 1, 2 in group 2 and 2 in group 3

**Supplementary Table 2**. Summary of susceptibility test outcomes by each trial treatment arm for the primary analysis population. MIC_50_ and MIC_90_; refer to the median and 90% quantile of isolates in the study respectively.

|  | **72 hour MIC (μg/mL)** | | |
| --- | --- | --- | --- |
|  | **Group 1**  **Amphotericin (n=92)** | **Group 2**  **Amphotericin and Flucytosine (n=96)** | **Group 3**  **Amphotericin and Fluconazole (n=88)** |
| **Amphotericin ^a^** |  |  |  |
| MIC_50_ | 0.512 | 0.512 | 0.512 |
| MIC_90_ | 1.024 | 1.024 | 1.024 |
| Geometric mean | 0.6089 | 0.6688 | 0.6692 |
| Interquartile range | 0.256, 1.024 | 0.2560, 1.024 | 0.256, 2.048 |
| **Fluconazole ^b^** |  |  |  |
| MIC_50_ | 8 | 8 | 8 |
| MIC_90_ | 16 | 16 | 16 |
| Geometric mean | 7.5846 | 7.2687 | 7.1085 |
| Interquartile range | 0.125, 512 | 1, 32 | 2, 32 |
| **Flucytosine ^c^** |  |  |  |
| MIC_50_ | 8 | 8 | 8 |
| MIC_90_ | 16 | 12 | 8 |
| Geometric mean | 6.1224 | 6.2586 | 6.6077 |
| Interquartile range | 0.96, 16 | 2, 64 | 2, 128 |

^a^Data were missing for 7 patients in group 1, 4 in group 2, 4 in group 3.

^b^Data were missing for 1 patient in group 1, 2 in group 2, 1 in group 3 at 72 hours.

^c^Data were missing for 1 patient in group 1, 1 in group 3 at 72 hours.

**Supplementary Table 3**

| **Classification of isolates as ‘fully sensitive’ or not as per CLSI suggestion**[3,4] | | |
| --- | --- | --- |
|  | **‘Fully sensitive’ strains**  **N** | **Other**  **N** |
| **Amphotericin^a^** | 173 | 103 |
| **Flucytosine^b^** | 114 | 162 |
| **Fluconazole^c^** | 213 | 63 |
| ^a^Fully sensitive = MIC ≤ 0.512µg/ml for amphotericin B  ^b^MIC ≤ 4µg/ml for flucytosine,  ^c^MIC ≤ 8µg/ml | | |

**Supplementary Figure 1.** Study enrolment and treatment assignment for our RCT of induction therapy for AIDS-associated cryptococcal meningitis in Vietnam [1]

**Supplementary Figure 2.** Distribution of antifungal susceptibilities of all isolates for amphotericin, flucytosine and fluconazole. The diagonal shows histograms of log2-transformed MICs, the panels below the diagonal display scatterplots (with jittering to avoid over-plotting) and the panels above the diagonal show Pearson rank correlations.

**
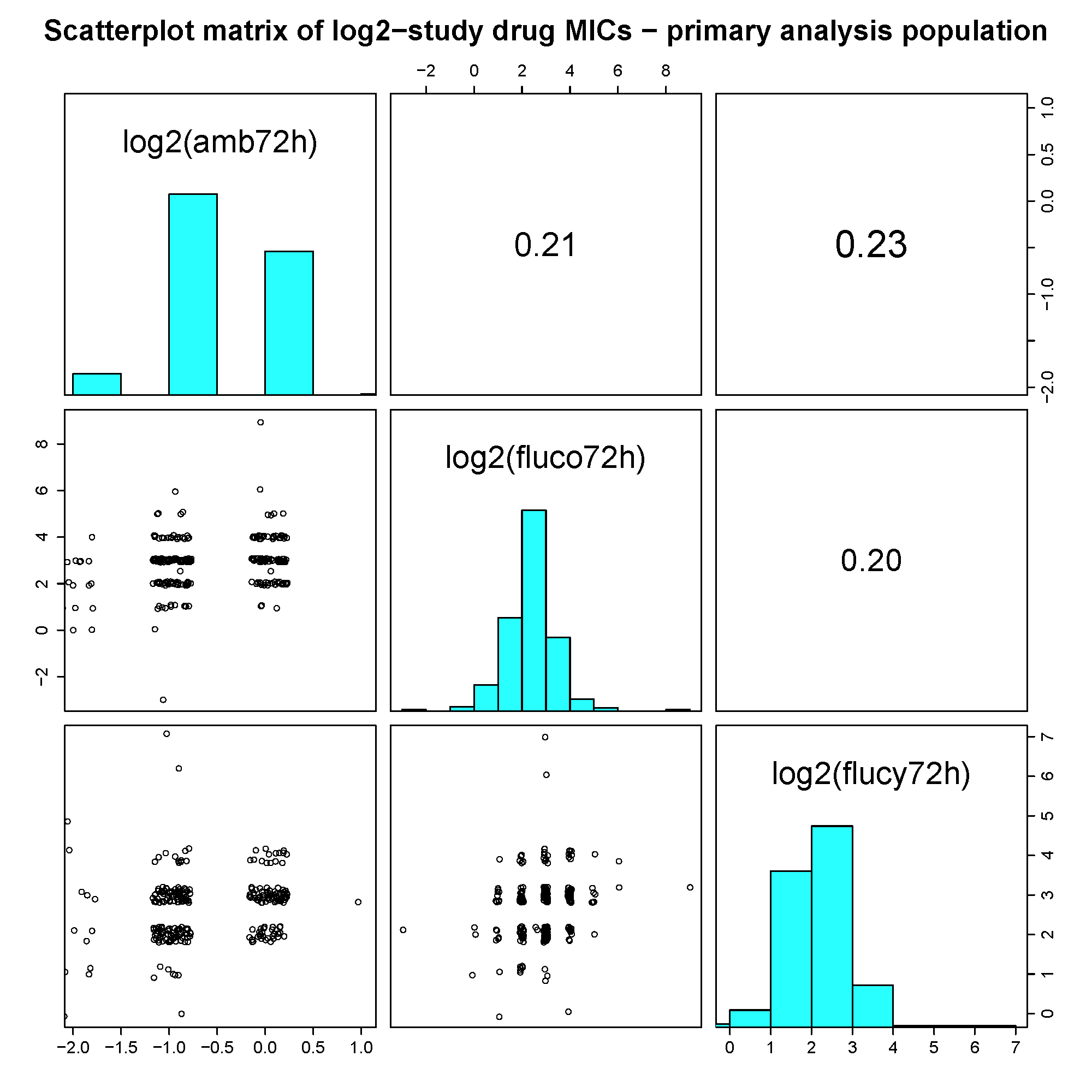
**

**Supplementary Figure 3.** Kaplan-Meier curves illustrating the estimated effect of antifungal susceptibility on time to death when comparing ‘susceptible’ and ‘non-susceptible’ isolates of *C. neoformans* for amphotericin susceptibility among patients receiving amphotericin monotherapy induction; flucytosine susceptibility among patients receiving flucytosine combination therapy induction; and fluconazole susceptibility across the primary analysis population and among patients receiving fluconazole combination therapy induction


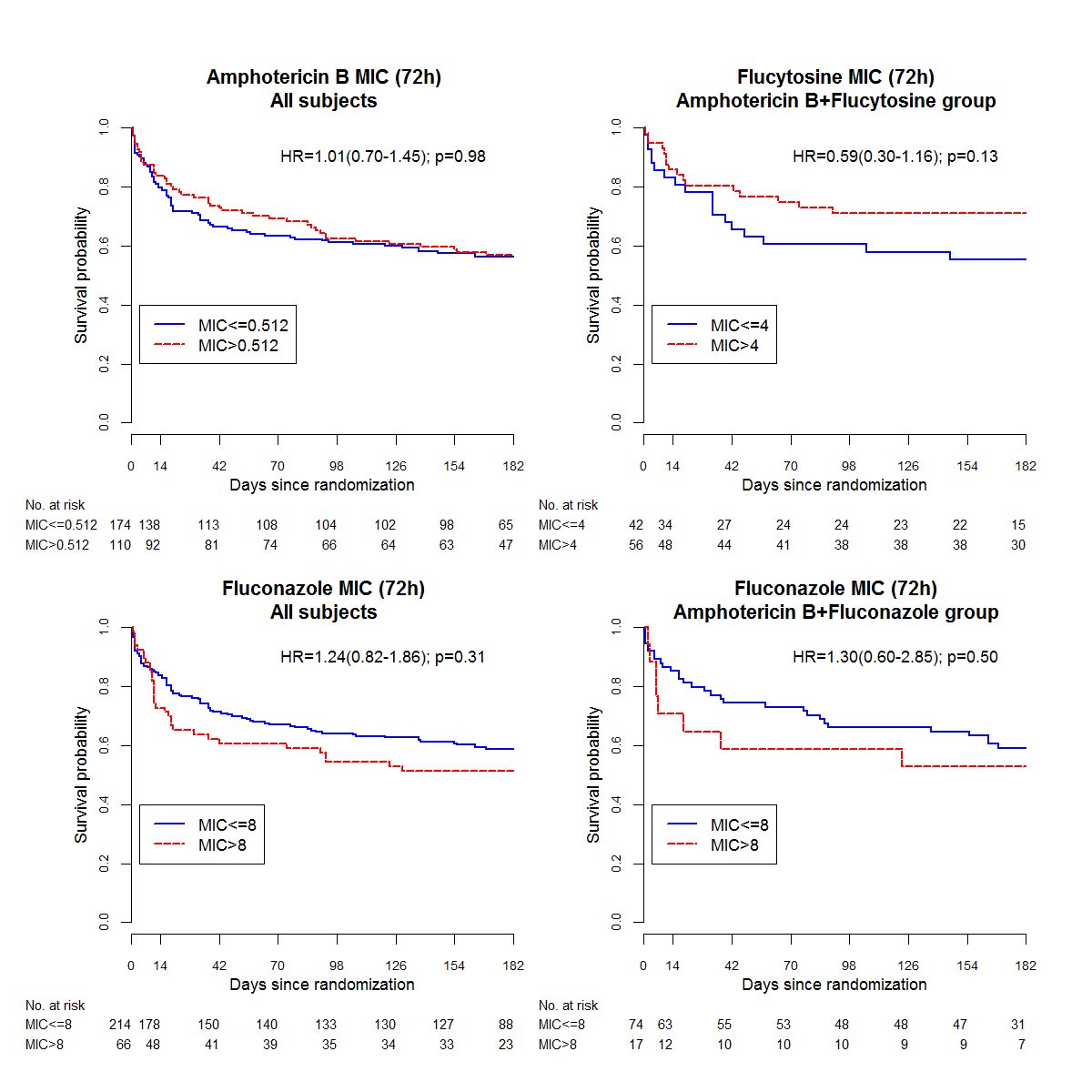


**References**

1. Day JN, Chau TTH, Wolbers M, et al. Combination Antifungal Therapy for Cryptococcal Meningitis. N Engl J Med **2013**; 368:1291–1302. Available at: http://www.pubmedcentral.nih.gov/articlerender.fcgi?artid=3978204&tool=pmcentrez&rendertype=abstract%5Cnhttp://www.nejm.org/doi/abs/10.1056/NEJMoa1110404.

2. Jarvis JN, Bicanic T, Loyse A, et al. Determinants of Mortality in a Combined Cohort of 501 Patients With HIV-Associated Cryptococcal Meningitis : Implications for Improving Outcomes. Clin Infect Dis **2014**; 58:736–745.

3. Clinical and Laboratory Standards Institute. Reference Method for Broth Dilution Antifungal Susceptibility Testing of Yeasts M27-A2. 2nd ed. Wayne, Philidelphia: 2002.

4. Arechavala AI, Ochiuzzi ME, Borgnia MD, Santiso GM. Fluconazole and amphotericin B susceptibility testing of Cryptococcus neoformans: Results of minimal inhibitory concentrations against 265 isolates from HIV-positive patients before and after two or more months of antifungal therapy. Rev Iberoam Micol **2009**; 26:194–197.
